# Social Norms Around Diet and Body Image: Evidence from Urban and Rural Vulnerable Groups in Colombia and Mexico

**DOI:** 10.3390/bs16050675

**Published:** 2026-04-29

**Authors:** Ana Cecilia Fernández-Gaxiola, Paula Veliz, Maaike Arts, Rowena Merritt, Ana María Narvaez, Anabelle Bonvecchio Arenas, Cássia Ayres

**Affiliations:** 1 Centro de Investigación en Nutrición y Salud, Instituto Nacional de Salud Pública México, Av. Universidad 655 Santa María Ahuacatitlán, Cuernavaca 62100, Morelos, Mexico; bonvecchio@insp.mx; 2 United Nations International Children’s Emergency Fund UNICEF Latin America and Caribbean Regional Office, Panama P.O. Box 0843-03045, Panama; pveliz@unicef.org (P.V.); marts@unicef.org (M.A.); rmerritt@unicef.org (R.M.); anamarianarvaezol@gmail.com (A.M.N.); cayres@unicef.org (C.A.)

**Keywords:** social norms, diets, adolescents, behavioral drivers, body image, vulnerable groups, Colombia, Mexico

## Abstract

In Latin America, the double burden of malnutrition is the region’s single most important public health concern for the incoming decade. Latin America’s burden of disease has distinct features in comparison to high-income countries: nearly 20 percent of NCDs are diagnosed in people under 60 years of age in Latin America, whereas only about 13 percent of people under 60 years of age in North America and Europe are diagnosed with these diseases. We aimed to better understand decision-making processes, preferences, and norms around food choices to provide input for future programming and policy suggestions at national and regional levels. We included key informant interviews and focus group discussions with parents and adolescents from urban and rural communities in three regions in Colombia and in Mexico. Results showed that food choices considered to be affordable, acceptable, accessible, and aspirational are driven by environmental and social factors that influence individual cognitive decisions. Across the study groups, cognitive biases influenced food decision-making in relation to eating out, natural, homemade, and “moderation”. At the sociological level, conversations, and social influences at home and in communities were strong indicators of dietary practices, health beliefs, and body size attitudes.

## 1. Introduction

Latin America and the Caribbean (LAC) have made progress at the regional level in reducing the number of children suffering from undernutrition, but large differences within and between countries make this an ongoing public health concern (Grajeda et al., 2019). The prevalence from a predominantly underweight population to a population with overweight and obesity has increased more rapidly in LAC than in other regions in the world (Hernández-Ruiz et al., 2022). LAC has also been particularly affected by the double burden of malnutrition, with a prevalence of 19.39% for mothers with overweight or obesity with at least one child with anemia, followed by a prevalence of 10.44% for overweight/obese mothers with at least one stunted child (Otten & Seferidi, 2022).

Traditional diets based on plants, whole grains, legumes, meat and fish are increasingly replaced with sugar, fats and oils, refined grains and ultra-processed foods. This transition appears to be the cornerstone of the region’s epidemic of nutrition-related noncommunicable diseases (NCDs) (Lustig, 2021). Compared to other regions, LAC has the highest rates of NCDs in the world. LAC’s burden of disease also has distinct features in comparison to high-income countries in North America and Europe: nearly 20 percent of NCDs are diagnosed in people under 60 years of age in LAC, whereas only about 13 percent of people under 60 years of age in North America and Europe are diagnosed with these diseases. Put simply, more people in LAC are getting sick from NCDs and they are getting sick at a younger age than people in high-income countries.

### 1.1. Social Norms and Food Behaviors

Food choice, food preparation, number of meals per day, time of eating, and portion size eaten make up food patterns and are an integrated part of cultural patterns in which each custom and practice has a part to play (Fieldhouse, 1995). Food habits are then established and maintained because these habits are practical or symbolically meaningful behavior in a particular culture and are hard to break, especially when attached to long-held cultural norms. These food habits are a product of the ecological forces, where the new changes in the food systems are embedded, and act within the context of historical conditioning and belief systems.

Social norms have a strong and regular impact on behaviors, but their force and form can only be soundly established through theoretical refinements (Cialdini et al., 1991). Social norms are influenced by a variety of contextual and structural factors, such as cultural norms, environmental factors, media influences, education, and peer pressure. These factors can complicate efforts to change norms and may require a multi-faceted approach in which policymakers, educators, and civil society actors, among others, play a role (WHO, 2021). There is evidence on social norms among adolescents, a particularly vulnerable group in terms of health behavior and body composition. Thus, to our knowledge, there is limited evidence on social norms around parents and communities, particularly in Colombian and Mexican population groups. While there are numerous studies on the epidemiological aspects of the double burden of malnutrition, there are fewer studies on the social life of eating as it relates to the region’s double burden of malnutrition.

### 1.2. Social Norms and Body Image

Social modeling is a major determinant of human eating behavior, whereby people use others’ eating as a guide for what and how much to eat, as parents’ model for children (Cruwys et al., 2015). Some eating behaviors related to body image, such as binge eating or fasting, with serious long-term health consequences, are also a growing concern (Neumark-Sztainer et al., 2006). Recognition of both the negative consequences and the relationship between social norms, diets and body image can help researchers and decision-makers to better understand people’s experiences of food and how they make decisions, which can inform policies and programs to improve population health, especially those in economic scarcity. A positive body image refers to having a healthy, accepting, and appreciative view of your body, while a negative body image involves dissatisfaction, shame or distress about one’s body. Although environmental factors shape body image, people with a positive body image also shape their environments in a growth-enhancing way, in a reciprocal process (Smolak, 2011). For example, they seek partners and friends that accept their bodies, also highlighting the relevance of socialization for the meaning of physical appearance. One’s body-focused experiences are particularly relevant among adolescence and young adulthood as these are crucial ages to educate on healthy food choices and improve nutrition and health in the long term.

### 1.3. The Behavioral Drivers Model (BDM)

The BDM (Petit, 2019) focuses its behavioral analysis on people’s individual and social behavioral interactions with others and their environment, as well as on how psychological factors influence people’s decisions and practices around food, eating and their bodies. We used the BDM to develop a holistic understanding about how people’s individual characteristics and socioeconomic contexts influence social norms around eating and body image. The BDM allowed us to consider the whole person or system, and not just the parts as the best way to understand and improve food and eating behaviors.

This study aimed to identify key social norms that influence behavior around diet, nutrition and body image among children and adolescents in Colombia and Mexico to better understand people’s decision-making processes, preferences, and norms around food choices and to provide recommendations for future programming and policy at both national and regional levels.

## 2. Materials and Methods

### 2.1. Study Design

This study used a participatory research to help focus on a process of sequential reflection and action (Cornwall & Jewkes, 1995).

### 2.2. Theoretical Framework and Definitions Used for This Study

We used a combination of definitions of social norms from Ajzen (2020), Lapinski and Rimal (2006) and Cialdini et al. (1991). Social norms refer to the collective normative beliefs among the study population. We referred here to norms that characterize the perception of what people think others do as “empirical expectations” and norms that characterize the perception of what people think others expect them to do as “normative expectations” (Bicchieri, 2005). This perspective emphasizes the process by which norms emerge, rather than their function or role in guiding group behavior. It, therefore, focuses on understanding the socially contingent knowledge and the ways that norms differ from other types of social codes and injunctions, such as legal and moral codes. Consistently, we used this lens to understand the empirical and normative expectations on body image (Bicchieri et al., 2011).

Meta-norms refer to a group’s processes for determining whether a norm violation has occurred (normative classification) and enforcing compliance. Meta-norms are essential to maintain normative social order because normative interactions occur in a noisy environment rife with perception errors and behavioral ambiguities (Hadfield & Weingast, 2012; Boyd & Mathew, 2021).

This study focuses on social norms as the unspoken rules of behavior and attitudes around eating, commensality and body image. This focus includes understanding what people actually and normally eat as a benchmark metric and then comparing that data against (1) how they talk about and conceptualize health, nutrition and body image, and (2) the context of their social and economic environments, which also impacts what is accessible, affordable and aspirational in their communities.

From this perspective, norms, like language, are created in coordination with environmental, sociological and psychological factors. This perspective emphasizes the process by which norms emerge, rather than their function or role in guiding group behavior.

Specifically, this study conceptualizes social norms as a type of social bias toward doing or condoning a given behavior. It defines social norms as occurring when two conditions are met. The first condition is the presence of a descriptive norm, which describes how people usually act, feel and think in each situation. The second condition is the occurrence of an injunctive norm, which describes how people think that they should behave, and which indicates that there is a social sanction for behaving differently from the way that most people behave in that situation (Cialdini et al., 1991). When both conditions are met, the behavior can be defined as a social norm.

The incorporation of the BDM aims to develop a “ground up” understanding of the social norms around food and commensality. To give readers and decision-makers a robust, contextual and processual understanding about what, why and from where social norms emerge around food and commensality, this study looks at how psychological, sociological and environmental factors interact to form social norms. This was done hoping to provide an understanding of the components that constitute norms, so that this document may hopefully serve as a guide to comprehend the regional landscape of social norms.

As the focus of the study is broad, we adopted a processual approach to unpacking the psychological, sociological and environmental variables in the conditions that produce the double burden of malnutrition in Colombia and Mexico. This approach emphasizes that there are numerous factors that lead to this population health outcome (Table 1), and that there are also numerous viable opportunities for promoting health in and with communities across these countries.

The strength of using the BDM lies in mapping out how the different drivers interact with each other to result in specific behaviors. It therefore provides a nonlinear, systems-oriented approach to unpacking the underlying patterns and causes of the ways that people eat and perceive their bodies. The BDM focuses its behavioral analysis on people’s social and behavioral interactions with others and their environment, as well as on how psychological factors influence people’s decisions and practices around food, eating and their bodies. Figure S1 demonstrates how these factors may interact in a decision-making pathway, i.e., to determine adoption of a behavior. The figure can be understood as a map that explains how individuals think and, consequently, how they decide to perform a certain behavior or not. It also shows how the different personal, social, internal and external factors can play a role in influencing behavior.

### 2.3. Country Settings

Colombia and Mexico have similar inequality and poverty conditions, conflict and economic conditions (Gabino, 2017), reflect North and South Latin American countries and sociocultural diversity, and their most vulnerable groups are experiencing the double burden of malnutrition (Grajeda et al., 2019). Yet the countries are confronting different stages of the food system transition, with Mexico being at a more advanced stage (Popkin & Reardon, 2018). In both countries, the capital, its surrounding, and nearby states were considered, to include urban (city and greater municipal), peri-urban and rural parts within each country.

### 2.4. Study Population

Based on BDM, we conducted (1) key informant online interviews with policymakers, advocacy groups, the education private sector and media informants to understand the environmental and sociological extrinsic factors (i.e., contextual, structural, and social elements and determinants); and (2) online focus group discussions with mothers and/or fathers of children 0–5 years of age, mothers and/or fathers of children and adolescents 5–19 years of age, and adolescents 14–16 years of age to understand psychological intrinsic factors (i.e., personal, individual cognitive elements and determinants). We focused on urban and rural vulnerable groups for whom the costs of poor health were outsized and had limited access to a variety of foods.

For the interviews, key informants were selected using snowball sampling and identified through our local data collection partner and local nutrition-focused organizations. Each stakeholder was selected based on the type of information that they could provide as it relates to the BDM. This approach produced holistic insights regarding social norms, nutrition, body image and metabolic health-related issues at the individual, community, and systems levels.

Inclusion criteria for informants included the following: (1) represent the public and/or private social sectors at the national level; (2) impact the behavior of children and adolescents by developing and influencing policies and programs around nutrition; (3) produce or hold sufficient and thorough knowledge on food and beverage industries and their impact on social norms and behaviors; (4) were accessible, had relevant information and were willing to share it; (5) had experience, capabilities and qualifications related to the objective of this study; and, (6) had the capacity to provide recommendations for policy design and implementation at the national and regional levels. Key informants were selected in order to comply with the interviews’ objectives (Table 2).

For focus groups, participants were selected using purposive sampling, aiming for equivalent gender representation, based on participants’ self-identification. Details were considered to allow for the disaggregation of data and triangulation with existing data (i.e., gender, age, urban/rural locality, and socioeconomic status). In Colombia, participants were from the departments of Antioquia, Boyacá, Bogotá, and Caldas, and in Mexico, participants were from the States of Jalisco, Mexico City, Oaxaca, Querétaro, and State of Mexico. While their experiences may be specific, there are numerous aspects of their circumstances that can be applicable and connected with those of other people across their country. Focus groups allowed the collective interpretation of social norms that guide behaviors around diet, nutrition and body image.

For focus groups, locations and participants were regionally/geographically representative, representing the sociocultural diversity of their country (in racial, ethnic and gender terms, among others), to enable the comparison of people’s experiences based on these characteristics. Participants belonged to similar socioeconomic groups and had similar relatable life experiences. In the case of adolescents, participants were of similar ages and tended to be older adolescents.

Due to social distancing measures during the COVID-19 pandemic, it was necessary for this study to be conducted online. To set this in perspective, in Colombia (World Bank, 2020a) and Mexico (World Bank, 2020b), about 60 to 70 percent of the national population has access to the internet, respectively. Focus group respondents were recruited from locations where access to the internet was widespread to prevent a selection bias. As a result, this study only reflects the experiences of people with internet access in the selected regions for this study. Yet we ensured that participants experienced financial vulnerability.

### 2.5. Data Collection

Instruments were developed with a focus on diet and nutritional behaviors and practices, by age group. Through simple questions, the different components of each BDM driver, the related diet, nutrition and body image social norms were systematically addressed (Bhandari, 2023). Table 3 shows the rationale for combining key informant interviews and focus groups under the BDM framework and the research tools used for data collection.

For questions on body image, an online questionnaire was designed that participants could answer independently, as this information was considered too sensitive to discuss in a group. To assess the perceptions of normative body image, specifically to understand how men and women talk about and conceptualize body image, participants reflected on the Stunkard Figure Rating Scale. The scale was developed to study how people perceive their body size and to investigate obesity (Stunkard et al., 1983). Data was collected from 14 March 2022 to 25 March 2022 in both countries.

The research team aimed to reach thematic saturation (Hennink & Kaiser, 2022) to make data collection robust and valid. To increase the validity of the findings, we triangulated these with existing, peer-reviewed data, as well as by including participants’ testimonies from key informant interviews and focus group discussions as much as possible (Flick, 2006). A literature review was performed to contextualize this study’s findings on social norms and triangulate our findings in relation to previously existing research.

All researchers and moderators were trained in steps and measures to ensure the quality of data collection and were provided with guidelines and protocols to ensure that the protection and dignity of respondents/participants were always maintained. All interviews followed the interview frameworks prepared to ensure that key areas of enquiry were captured, to allow for thematic analysis across data sources, as well as for participants to guide the direction of each interview according to their priorities (Supplementary Materials S1). All key informant interviews were audio-recorded with previous informed consented from participants and transcribed verbatim. Researchers took notes during the focus group discussions. Informed assent of this study’s participating adolescents took place after their parents and legal guardians gave their consent for adolescents under their responsibility to participate (Supplementary Materials S2).

### 2.6. Data Analysis

Interviews and focus groups were transcribed verbatim and reviewed and edited for accuracy. Transcriptions were analyzed following a thematic approach identifying key themes and codes, using a primarily inductive and semantic focus (Braun & Clarke, 2024). A priori codes were predefined by the different drivers and themes within each driver of the BDM, guiding coding in a systematic way. During the codification, the different social norms that emerge in each theme were coded as relevant and/or repetitive ideas. Achieving full understanding of the codes was considered an indicator of saturation across data collection tools used in each country (Hennink et al., 2017). We heard very similar suggestions for effective content after 10 key informant interviews and 12 focus groups with different stakeholders, both in Colombia and Mexico. These codes were then grouped to create a perceived social norm variable within each of the BDM drivers. Internal coherence between themes and distinction between them was discussed and agreed upon.

Two researchers analyzed the information and compared coding. Both the audio recording and the assistant moderator’s notes were reviewed along with the translated transcripts of the interviews. Findings were organized according to the BDM and divided into psychological, sociological, and environmental drivers, integrating information from focus group discussions and stakeholders’ interviews in the narrative as needed and organized according to the main elements that this study identified as influencing social norms.

## 3. Results

### 3.1. Key Informant Interviews

A total of 10 interviews were held with policymakers and other key stakeholders at the national and local levels and included representatives from government ministries, international and national agencies, non-governmental organizations, civil society, child nutrition experts, and community leaders, in each country (Table 4). These interviews provided a holistic understanding of the policy and structural environment of each country.

### 3.2. Sociodemographic Characteristics of Focus Group’s Participants

#### 3.2.1. Participant Details: Colombia

In urban areas, mothers (n = 18) were 30 years old on average and, in general, had completed a higher degree compared to fathers (n = 11). In rural areas, mothers (n = 6) and fathers (n = 11) had similar ages (37.5 and 36.8 years on average, respectively). Vulnerability factors, such as changing residence due to violence and/or political economic reasons, were more frequent among parents in urban areas compared to parents in rural areas. Only one person in this study reported being a member of an indigenous community. Adolescents in rural areas were more likely to have one more year of schooling than their urban counterparts. Adolescents in urban areas were more likely to have experienced forms of socioeconomic vulnerability compared to their rural counterparts (Table 5).

#### 3.2.2. Participant Details: Mexico

In urban areas, mothers (n = 14) and fathers (n = 14) had varying degrees of education. The average age of mothers and fathers in urban and rural areas was similar (i.e., 35.3 years and 35.5 years, respectively). Most vulnerabilities parents experienced were economic. Likewise, the size of households in both urban and rural areas were similar (3.6 to 4.5 persons). In urban areas, most mothers had completed high school education, while most fathers had completed only middle school. About a third of participants in urban areas and less than a third in rural areas experienced unemployment. Only a few families in urban areas received government support.

Adolescents in Mexico experienced a range of socioeconomic vulnerabilities. In urban areas, most female and male adolescents had completed middle school. In rural areas, nearly all female adolescents reported middle school as their highest level of attained education, while half of male adolescents reported middle school (Table 6).

### 3.3. Social Norms Around Diet and Body Image from Focus Groups and Interviews in Colombia

Most participants from urban communities shared that they eat about three meals per day but did experience some form of food insecurity as a reduction in the variety and/or quality of their diets, while many participants in rural communities described having only two meals per day because they could not afford more meals. Food insecurity may have been aggravated by the COVID-19 pandemic, but it was not specifically discussed. Some parents receive governmental support to buy groceries; most people do not. Urban households eat a variety of foods, including fruits, vegetables and protein- and carbohydrate-rich foods (e.g., in the form of an arepa and/or potato), as well as sweetened foods and beverages.

In rural communities, most participants described having only two meals per day because they could not afford more meals. Parents of children in rural communities in government-run pre-primary schools shared that they are reliant on school meal programs, which include breakfast, lunch, and a snack, as a type of economic support for families with young children. Despite the reduced number of meals, most people in the study eat a variety of predominantly unprocessed foods. Breakfast in some regions typically includes foods like arepa, a type of maize patty that, like a sandwich, is often filled with cheese or eggs; or they eat a different combination of a carbohydrate-rich and a protein-rich food, e.g., rice and chicken.

#### 3.3.1. Psychological Drivers in Colombia

Parents and adolescents both were knowledgeable about overall nutrition, especially about including /providing their children with fruits and vegetables. Conversely, their knowledge about the impact of sugar-sweetened beverages (SSBs) is varied. Cognitive bias was identified from participants, considering “natural” sugar, e.g., in juice and panela water, to be nutritious. This delineation between “natural” and “artificial” foods contributes to salience bias, where the negative effects of consuming foods and diets rich in sugar appear less obvious because some sugars are considered natural and therefore healthy. Participants in urban areas often felt less capable of being able to afford and include a wide variety of fruits and vegetables in their diets, compared to participants in rural areas. This demonstrates a structural barrier but also a lack of confidence (self-efficacy) on their capacity to control their diets. Participants in urban areas also referred to feeling less capable at changing their habits of drinking SSBs. This finding also reflects a lack of self-efficacy in their ability to change their health behaviors.

Other cognitive bias was identified from the evoked positive memories of spending time with family when eating out at international restaurant chains. The positive and sentimental recollections of time spent with family (nostalgia effect) can drive the decision to eat certain foods in the present. Among adolescents, eating out and buying food at convenience stores often evoke positive emotions and feelings of social independence. The positive associations of independence through items that adolescents can afford can drive their purchase of ultra-processed foods at convenience stores.

Participants considered balancing and moderation of consumption of “artificial” foods with a wide variety of “natural” foods important to achieve and maintain good health, although people’s definitions of moderation varied. An intent factor was identified when participants shared that health is predicated on what a person eats and that it involves both physical and emotional wellbeing, rather than a particular body size. This reflects that those participants regarded consuming nutritious foods and being emotionally well as an intention of health. Participants considered affordability and quality, which they defined as “wholeness” (e.g., unbroken rice grains), the most important factors of food purchase decision-making. This showed the intention to strike a balance between affordability and quality as the desired outcome of food decision purchases.

The following word cloud displays the most common words that participants used to describe healthy behaviors: “physical activity”, “water”, “diet”, “food”, “balanced eating”, “junk food”, “sports”, “exercise”, “rest”, “sleep”, “fruits” and “eating healthy”.

#### 3.3.2. Sociological Drivers in Colombia

Most adult and adolescent participants in Colombia in rural and urban areas tend to avoid eating snacks; thus, these are given to young children as they believe their diets should not be as restrictive as their own, and they all eat a wide variety of foods, mainly animal products, and fruits and vegetables when possible. Social influence was also identified in participants’ habit of consuming SSBs around toddlers, which contributes to their curiosity and interest in trying SSBs as toddlers. The beverages that they drink throughout the day include hot chocolate, panela water (i.e., water with molasses) and coffee in the morning, and cold panela water, fruit, and flower-infused water, as well as juice and SSBs, during the rest of the day. Only a few people reported drinking plain water regularly. The descriptive norm of drinking SSBs is driven by both environmental, as they are widely available and marketed, and by sociological factors. Many people also consider drinking SSBs as a sign of economic wellbeing, as these are seen to indicate that these people can afford non-essential items. People in rural areas, more than in urban areas, view these drinks as a sign of a person’s economic stability.

These findings emphasize that socioeconomic and environmental factors drive the development of drinking SSBs as a social norm in Colombia. Most people, however, consider SSBs to be unhealthy and make clear distinctions between “natural” and “artificial” foods and beverages, and natural foods are considered healthy. For this reason, most people consider juice and cane sugar to be healthy, and they add it frequently to their beverages. While this metric is often useful, it can skew their perception about the healthfulness of SSBs, such as sweetened juice and *panela* water. This demonstrates that discursive definitions, e.g., between the natural and the artificial promoting eating more “natural” regardless of the food, can constitute social norms that impact decision-making.

Conversations about health that participants have with family members are the most important vectors of information and form of social influence impacting people’s knowledge, attitudes, and practices around health: first, because the home is an important socialization and education place; second, because of children’s desire to conform with family members.

Eating out is reserved for celebration and occasional recreation based on financial resources, and in both urban and rural communities, it is associated with economic wellbeing and showing affection. It also shows a community dynamic where eating out symbolizes economic stability and facilitates family and social cohesion. The greater initiative that men—as opposed to women—take to invite their families out to eat indicates that there is a gender norm where men are people who make the decision to eat out and spend money.

Gender norms in people’s environments are also driven by social influence factors. While most people describe household labor as the responsibility of women, food serving in urban households is considered a family activity. Most men and male adolescents refer to their participation as supportive rather than as their responsibility. This discursive formulation both drives and underscores this norm: it reinforces the belief that household labor is women’s responsibility but an optional activity for men. Some fathers said that they participate in meal preparation when they are unemployed, to support their households and wives. This finding further underscores the strength of this gender norm, as far as it demonstrates that economic exigency compels them to participate.

Body size responses were also driven by social factors in people’s environments. Most respondents considered thin bodies healthier than larger bodies in both urban and rural communities, which contributes to the meta norm, or belief, that thinness is demonstrative of health. Men observe a wider range of sizes in their communities, while most people said that, also in their communities, women’s bodies tend to be larger. Participants consider sizes 3 to 5 to be “healthy” for women but tend to rank sizes 5 to 7 as “normal” for women. This descriptive norm was driven by people’s conception of health, which they described as being determined by exercise and “healthy eating,” as well as by rest.

#### 3.3.3. Environmental Drivers in Colombia

Structural factors in people’s environments also impact diet norms and health outcomes. Infrastructural, socioeconomic, and regulatory factors create health precarity—vulnerability—for many people in Colombia, and these social determinants impact people’s health in inequitable ways. At the socioeconomic level, factors include inflation of food prices; access and cost of water; and cultural and linguistic marginalization, which creates barriers to accessing social, public and health services. Moreover, the implementation of short-term health policies that are contingent on the support of a rotating presidential administration, politicizing public health, leads to inconsistent messaging and policy, and creates barriers to developing and implementing stable, long-term policies. Short-term policies contribute to the instability of health programs, irregularities in the nutrition messages communicated to the public and inconsistencies in the types of programs that exist and the programs and policies that are funded.

High food prices (economic barrier) limited most participants’ ability to buy more than their essential carbohydrate- and protein-rich foods and decreased their ability to afford a wide variety of produce.

Limited transportation infrastructure across the country impacts both small and large businesses’ ability to transport perishable foods from rural and peri-urban areas. Another infrastructural barrier is created by insecurity and political conflict, which hinder the government’s ability to conduct health and nutrition programs or reach participants in some parts of the country.

Finally, structural factors, such as changes in the food landscape driven by large-scale industrialized agriculture and the globalization of processed foods, lowered prices of processed foods and increased their variety, availability and affordability. These changes create a choice environment where both nutrient-rich and nutrient- poor foods are accessible. At the same time, geographic remoteness and the cultural, ethnic, and linguistic marginalization of people from indigenous and Afro-descendant communities create barriers to accessing competent and localized health, social and public services. These factors contribute to an overall environment where some people are predisposed to worse health and social outcomes than others.

### 3.4. Social Norms Around Diet and Body Image from Focus Groups and Interviews in Mexico

In rural Mexican communities, much like in urban areas, both adolescents and adults described consuming high amounts of sugar and low amounts of fiber-rich foods. Virtually all respondents described eating three meals, which consist of breakfast, lunch and dinner, plus an afternoon snack. The foods that they eat, particularly for snacks, include SSBs, cookies and sweetened cereals, as well as meat and bread. Breakfast typically includes carbohydrate-, fiber- and protein-rich foods like breakfast cereal, fruit, eggs, beans and local foods such as enfrijoladas and entomatadas. *Enfrijoladas* are a type of enchiladas made from dipping corn tortillas in a sauce of coarsely pureed black beans, folding them in quarters and heating them on a griddle. In comparison to people in urban communities, participants in rural areas described eating a much wider variety of fruits and vegetables. Participants described usually having three meals per day, although, in rural areas, some referred to having only two meals per day and perceiving food insecurity.

Notably, the swampification of food environments (i.e., a spatial metaphor to describe neighborhoods where fast food and junk food inundate healthy alternatives) (Rose et al., 2009) interacts with behavioral and sociological factors that influence the foods that people choose to eat and that contribute to normative behavior and practices.

#### 3.4.1. Psychological Drivers in Mexico

One of the themes that many participants shared is that “moderation” regarding unhealthy foods is critical to achieving health. Similar to participants in Colombia, when participants were presented with a choice, they did not exercise such moderation. For example, when talking about the choice of drink, participants in rural communities across age groups referred to carbonated SSBs as being indispensable parts of their diets and social identities. Parents expressed concern about promoting moderation in food and beverage choices among their older children and adolescents, even though they as parents have a wide range of understandings about what moderation means. Several parents said that they do not drink carbonated SSBs frequently, while sharing that they drink these beverages 1–3 times per week. This suggests they may consider drinking carbonated SSBs daily to be frequent, and thus base their understanding of moderation on such frequency, and/or a tendency to overestimate one’s control over decisions made on impulse, which influences individuals’ decision-making. Other behavioral findings, different from Colombian participants, were that habit influences what people eat to fill “cravings.” The tendency to eat sugar-sweetened or sodium-rich foods for snacks demonstrates that this practice is a habitual way for participants to satiate cravings.

The widely held definition that equates “homemade” with healthy foods and store-bought with unhealthy ones contributes to many participants considering homemade sugar-sweetened foods and beverages (e.g., sugar-sweetened flavored water and gelatine) as nutritious. These findings show that discursive constructions create and influence normative beliefs about the nutritional qualities of foods.

Like participants in Colombia, many Mexican participants have positive associations with eating out: as a form of leisure and recreation with family; as a reward to oneself or a loved one; and as an expression of care and love. These positive associations with eating out contribute to a nostalgia effect where positive memories of the past drive impulses, habits, and desires in the present. Adolescents, especially male adolescents, often associate eating out with friends and/or buying foods at convenience stores by and for themselves with social independence. This demonstrates a type of affect bias because the positive associations of independence can drive adolescents’ purchasing of food vendors and convenience stores.

Like adults in Colombia, Mexican adults in urban areas frequently describe feeling incapable of changing the socioeconomic circumstances affecting their ability to buy more vegetables. While this reveals a structural barrier, it also reflects an emotional experience where participants feel disempowered, or lack in self-efficacy, regarding their ability to change their health consequences. Most participants in this study regularly experience financial scarcity and food insecurity, and they regularly limit their food intake to essential items, which are often protein- and carbohydrate-rich foods.

Different from participants in Colombia, many adults in rural areas, and occasionally in urban regions, describe growing food at home to save money and increase their consumption of fruits and vegetables. Their feeling of capability, or self-efficacy, to increase the availability of fruits and vegetables at home is driven by their knowledge of and greater opportunities for gardening in rural areas. For many participants, gardening is a familiar practice that can be shared (taught, learned) to grow better foods locally. Growing and sharing local foods are ways in which people build community and relationships.

The following word cloud displays the most common words participants used to describe healthy behaviors: “drinking water”, “having a balanced diet”, “sleep”, “exercise”, “avoid excesses, fats”, “junk foods”, “eating fruits and vegetables” and “eating healthy”.

#### 3.4.2. Sociological Drivers in Mexico

Like in Colombia, Mexican adults, adolescents, and older children’s habit of consuming carbonated SSBs around young children contribute to the interest in and curiosity to try these drinks, resulting in a social influence on children’s desire to try them. In many families, people learn to consume carbonated SSBs (either plain, with lemon and/or baking soda, or boiled into a syrup) in remedial ways, e.g., when they feel feeble or have indigestion, showing that household practices are a form of social influence that drives behavior. The family is the first context where participants acquire health knowledge and the home is the place where knowledge about disease and disease prevention becomes salient, e.g., through the illness of a family member.

Gender norms identified included the expectation on women and female adolescents to be responsible for household labor and meal preparation, which indicates that there is a gender norm where women face this expectation while men and male adolescents do not. This converges with findings in Colombia. Likewise, the greater frequency by which men take the initiative and make the decision to treat their families to eat out shows a gender norm where men are more often the ones who make decisions about recreation and spending. Moreover, several men said they like to give their wives a break from housework, such as preparing and serving food on Sundays, by taking them out to buy food at food stands. This underscores the strength of the gender norm that meal preparation is considered women’s work.

Finally, while most participants have larger bodies in the communities where they live, the latter consider thinner bodies to be healthier than larger bodies, evincing a meta-norm in which thinness is considered demonstrative of good health.

#### 3.4.3. Environmental Drivers in Mexico

Drinking SSBs is a norm in both urban and rural areas, driven by the environmental availability of these drinks and their popular association with wellbeing. Infrastructural factors like lack of consistent access to potable water and the higher cost of bottled water in comparison to industrialized beverages likely contribute to this norm. However, different from the findings in Colombia, Mexican participants tended to focus on the ways that they associate SSBs in general with remedial uses, which shows that this association is the main social driver of this norm. For example, several mothers described making sugar-sweetened, fruit- and flower-infused drinks as an act of care for their families. Many people also associate drinking carbonated SSBs with a reward for hard work. Many people have strong brand preferences and generally prefer to drink one or two brands rather than a variety of branded beverages. Particular to the Mexican children was their description of regularly consuming carbonated SSBs, which they typically start drinking around the age of two or three years.

Parents expressed that they often feel excluded and reprimanded by school educators when implementing nutrition programs because they are told that the snacks they provide their children are not allowed. This finding is unique to Mexican participants and reveals that the communication environment at schools and the implementation of school regulation can make parents feel blamed and reprimanded for feeding their children certain types of snacks. Adolescents expressed that they often find school nutrition education a memorable experience in whose programming they feel included, rather than excluded or reprimanded. Conversely to parents’ experiences, this finding shows that when aimed at students, the school communication environment makes them feel welcome and included in conversations about health.

Changes in Mexico’s food landscape driven by free trade agreements (e.g., NAFTA and USMCA) have shifted consumer diets and increased the availability and affordability of processed foods. These changes to Mexico’s food landscape are an infrastructural driver of behavior because they create a choice environment where both nutrient-rich and nutrient-poor foods are accessible and affordable. Other infrastructural barriers are the lack of green spaces, crime-related insecurity, high concentration of street vendors, limited infrastructure for active commuting (e.g., cycling and walking), and the long distances that people commute to reach their workplace or home in the cities, which make it difficult to increase physical activity. These findings were unique to Mexican participants.

A consistent theme in conversations with stakeholders is the numerous policies on paper and the gap between nutrition policies in practice. This finding shows that among governing entities, there is a lack of a consistent, coordinated, and multi-sectoral approach when implementing policies. Informants referred to structural factors such as free trade agreements, e.g., the former North American Free Trade Agreement and the current United States–Mexico–Canada Agreement, which have transformed Mexico’s agricultural economy toward industrialized monoculture, industrialized food and lowered rates of local food production.

## 4. Discussion

Our study documented personal experiences around diet and body image from a variety of participants with consistent use of the BDM to interpret cognitive biases, self-efficacy, social influence and structural barriers. Our findings show that food choices in both countries’ participants were determined by environmental and sociocultural factors that define foods as affordable, acceptable, accessible, and aspirational.

### 4.1. Psychological Drivers in Both Countries

At the psychological level, the most frequent drivers of food choice among our participants included cognitive biases and lack of self-efficacy. Across the study groups in Colombia and Mexico, we found that cognitive biases influence food decision-making, particularly in relation to eating out, natural, homemade, healthy foods, and “moderation.” Although cognitive bias is recognized as a factor influencing food choices and intake (Provencher & Jacob, 2016; Lev-Ari et al., 2021), participants often described natural as always better and healthier, but this cognitive bias is not limited to the study participants, neither is it a unique perception within these countries. Food branded with a “natural label” can be found in any grocery store in the US (with no clear definition of “natural”), and consumers consider this label an important attribute when making a purchasing decision (Goodman, 2017).

Body image concerns related to weight or other dimensions of appearance are now prevalent on a global scale due to their prevalence and association with poor mental and physical health (Rodgers et al., 2023). Participants in Colombia and Mexico shared similar attitudes about body image and size, which suggests a common norm in these vulnerable settings. However, in the context of an accelerated highly visual culture, facilitated by digital technologies in recent years, which have exacerbated different attitudes related to the body’s appearance with health, power, and privilege (Bourdieu, 2018), interventions to mitigate these concerns at the individual and systemic levels are needed, particularly targeting children and adolescents as “digital natives” (Yoo, 2021; Gualdi-Russo et al., 2022).

Many of the psychological mechanisms described in our study (e.g., moderation rhetoric, naturalness bias, social modeling, thinness-as-health norms) are well documented in experimental and survey research—often in high-income, Western samples (Biltekoff, 2013; Saguy, 2013; Bicchieri, 2005; Puhl & Heuer, 2009; Meier et al., 2019; Cruwys et al., 2015), and our study demonstrates that these mechanisms are embedded in everyday discourse in vulnerable Latin American contexts.

### 4.2. Sociological Drivers in Both Countries

At the sociological level, the most frequent drivers of food choice among our participants included conversations and social influences at home and in communities. These were strong indicators of the dietary practices and the attitudes about body size that people have, as well as of the beliefs about health that they hold. At the individual and social levels, as people adopt similar habits and ways of thinking about food and health, these attitudes become shared social norms within families, communities, and groups of people. Accordingly, individuals with high self-confidence and self-control are more likely to resist peer pressure and make decisions that align with their values and goals (Shah & Asghar, 2023).

In addition to behavioral factors, the social influence of participants’ families is an important way in which people adopt habits and learn about health. Family is well placed to influence wellbeing, being one of the significant contributors to an individual’s health status (Ho et al., 2022). For example, through modeling health behavior and providing support for improving wellbeing, the remedial uses of SSBs and the association of homemade beverages with wellness were often learnt within the family context among Colombian and Mexican participants. Plus, children are mostly passive recipients of this health influence. Thus, changing the norms and behavior patterns in a social unit like the family may create longer-lasting and larger-scale behavioral change (Secker-Walker et al., 2000).

### 4.3. Environmental Drivers

At the environmental level, the most frequent drivers among our participants included structural barriers. The environments in which participants live facilitate decisions to eat ultra-processed foods. Consistent with other studies (E. Pineda et al., 2021; A. M. R. Pineda et al., 2023), the predominant food systems in these two countries (i.e., agro-industrial production, monoculture, exploitation of natural resources, food distribution dominated by multinational supermarket chains, essentially in large urban centers, including food labeling regulations, among others) generate a significant negative environmental impact. Also consistent with other studies (Bauman et al., 2012), the built infrastructure, from roads to sidewalks, and structural factors of food policies and regulatory frameworks create barriers in accessing nutrition-rich foods and exercising regularly.

Environmental factors, including infrastructural, socioeconomic, and regulatory factors, are also important in affecting the accessibility and affordability of foods and the ways that people conceptualize health. Infrastructural factors, such as limited road infrastructure, political insecurity and the privatization of natural resources, can negatively impact local food production and transportation. There is an increasing concern over structural factors that promote unhealthy dietary patterns and undermine the adoption of healthy eating, and advocating for structural changes plays a central role in forging more healthful, sustainable, and equitable food systems and environments.

### 4.4. Implications for Programming and Policy

By using triangulation and different research tools to study the same phenomenon to see whether the findings converge, our study represents a cross-method validation. Triangulation also enhances validity and credibility and produces more robust conclusions (Flick, 2018). Both psychological and sociological mechanisms were reflected in lived narratives in different cultural settings and structurally constrained environments of our participants, an important contribution of our findings (Denzin, 1978; Creswell, 2014).

Our findings reflect the experience of study participants in the selected regions with access to the internet yet who experience financial vulnerability, which were our explicit conditions. These findings thus aim to represent the experiences of this sub-population, rather than those of the national population. Our study reveals how and why norms emerge, uncovering meaning, mechanisms and social dynamics, which are crucial in areas shaped by norms and expectations that are not directly observable in controlled settings (Charmaz, 2004; Bicchieri, 2005).

Due to the COVID-19 pandemic, participants were all interviewed online as opposed to face-to-face. The pandemic may have also influenced perceptions of certain foods and their healthiness or unhealthiness. Finally, focus group discussions and key informant interviews also present a study limitation; even as they add depth of insight, they can limit the study’s findings if participants feel a social pressure to conform to the norms of the group. Hence, similarities emerged in perceptions and social norms between participants in Colombia and Mexico, and the triangulation of information confirmed the relevance of the findings. Despite these limitations, the authors felt that sufficient information could be retrieved to inform these vulnerable groups’ experiences.

## 5. Conclusions

Our findings add to the understanding of social norms around diet and body image in Colombian and Mexican vulnerable population groups in urban and rural areas and stakeholders. Drivers and barriers were linked to factors such as health, wellbeing, gender, self-efficacy, power, economy, regulations, and infrastructure. The types of conversations that people have about diet and health with family members and people in their communities are a critical form of social influence that impacts how they conceptualize and understand food and health.

Despite the growth of academic understanding of the importance of the social determinants and drivers of diet and health, decision-makers do not always incorporate the role they play in shaping diet and health. As a result, the social determinants and drivers are seldom considered in many decision-making processes, so they fall short of properly addressing them. Social norms can confront the societal, environmental, and health challenges our societies face, and this study provides a better understanding of how these emerge and evolve in place. There is an urgent call to address these social determinants and drivers, as they are the root causes of inequity.

Our study does not extend the BDM theoretically, but it demonstrates its practical utility in complex, real-world settings, particularly when integrating stakeholder interviews with family and adolescent focus groups. This triangulation across actors and ecological levels strengthens internal coherence and policy relevance.

## Figures and Tables

**Table 1:** Summary of factors and definitions by BDM driver.

BDM Driver	Factor	Definition
Psychological insights	Salience bias	A focus on items of information that are more prominent while ignoring items that are less noticeable.
	Nostalgia effect	The influence of sentimental feelings of the past on present and future decisions.
	Affect bias	The influence of current emotions on rapid decisions.
	Self-efficacy	Belief in one’s own capability to change one’s behaviors to reach a specific goal.
	Intent	Goal or purpose in an action or value.
	Interest and attitudes	The significance of a characteristic and the perspectives around it.
Sociological insights	Social influences	Intentional and unintentional efforts to change a person’s behavior.
	Community dynamic	Social and environmental characteristics of a group of people.
	Gender norms	Principles governing the behavior of adults and children to practice conducts considered appropriate for their gender.
	Meta-norms	Collectively held beliefs and values that facilitate group cohesion and social order.
Environmental insights	Communication environment	Social setting in which many different narratives, beliefs and attitudes interact.
	Governing entities	Administrative and regulatory organizations recognized by a community.
	Infrastructural factors	The set of facilities and systems that impact the functionality of an economy and people’s participation in it.
	Structural factors	Obstacles (policies, practices, norms) that impact a group disproportionately and contribute to social disparity.

**Table 2:** Interviews’ objectives per stakeholder.

Stakeholder	Objectives
Government	Assess knowledge, attitudes and commitment of policymakers toward addressing the epidemic of overweight and non-communicable diseases in children, adolescents and parents who are affected by nutritionally poor diets and negative body image. Identify current and former policies and legislation around diet and image.
Civil society organizations	Understand the perspective of advocacy groups and community leaders in terms of social norms that influence behaviors (diet and body image of children and adolescents) and the challenges and opportunities toward promoting social and policy change.
Education sector	Understand the perspective of teachers and staff around social norms that influence children and adolescents’ behaviors.
Private sector	Identify attitudes and commitment in relation to the application of measures to prevent and/or control unhealthy behaviors around diet and body image of adults, children and adolescents.
Media	Identify contents used to disseminate information on health and nutrition (aspirational narrative and discourses).
Academia	Confirm current trends on social and behavioral change around nutrition and positive body image.

**Table 3:** Behavioral Drivers Model (BDM) by stakeholders and research tools.

BDM Level—Stakeholders					Research Tool
Environmental	Policies	Legislation		Media	Key informant interviews
Sociological	Education sector		Community leaders		Key informant interviews
Psychological	Parents		Children/Adolescents		Focus groups

**Table 4:** Key informants interviewed in Colombia and Mexico.

Country	Policymakers	Education Sector	Civil Society Organizations	Private Sector	Media	Academia
Colombia	2	2	2	2	1	1
Mexico	2	2	2	2	1	1

**Table 5:** Sociodemographic characteristics of focus group’s participants from Colombia.

	Urban Context				Rural Context			
	Mothers	Fathers	Female Adolescents	Male Adolescents	Mothers	Fathers	Female Adolescents	Male Adolescents
Number of participants	18	11	7	7	6	11	7	8
Average age	30	36	14.8	15.1	37.5	36.8	15.5	15
Highest degree education *	HS (14) and TP (4)	MS (4), HS (84) and TP (3)	MS	MS	MS (1), HS (4), and TP (1)	5th grade (1), MS (2), HS (7) and TP (1)	MS	MS
People living in participant’s household	4	3.4	4	4.1	3.8	1.8	4.1	2.8
	**Household income**							
Col $700.00/mo.	13	5	4	6	3	4	1	4
Col $700.00 to Col $1 million/mo.	5	6	3	1	3	7	6	2
Col $1 million to Col $2.8 million/mo.	0	0	0	0	0	0	0	0
	**Vulnerability factors**							
Belongs to an indigenous group	1	0	0	0	0	0	0	0
Experienced religious/belief discrimination	5	1	1	0	0	2	0	0
Gender and sexual diversity	13	4	3	6	2	4	1	0
Disabilities	8	2	0	2	2	2	0	0
Out of work + 3 mo.	9	6	4	3	4	4	1	0
Government program benefits	10	3	3	5	1	4	3	2
Remittances	0	0	0	0	0	1	0	0

* TP = Technical Professional; HS = High School; MS = Middle School.

**Table 6:** Sociodemographic characteristics of focus group’s participants from Mexico.

	Urban Context				Rural Context			
	Mothers	Fathers	Female Adolescents	Male Adolescents	Mothers	Fathers	Female Adolescents	Male Adolescents
Number of participants	14	14	7	6	13	12	6	6
Average age	35.3	35.5	15.1	14.6	32.23	38.3	15.3	15
Highest degree education *	ES (1), MS (11) and HS (1)	MS (11) HS (1) and UD (1)	ES (1), MS (5) and HS (1)	MS (5), HS (6) and UD (3)	MS (3), HS (6) and UD (3)	ES (1), MS (2), HS (1) and UD (8)	MS (5) and HS (1)	MS (3) and HS (3)
People living in participant’s household	4.3	3.6	4.14	4	4.3	3.6	4.5	4.16
	**Household income considering the earnings of all people living in the same household**							
Mex $2700 mo.	0	1	1	1	0	0	0	0
Mex $2700 to Mex $6799 mo.	14	10	5	5	9	4	3	3
Mex $6800 to Mex $11,599 mo.	0	2	1	0	4	8	3	3
	**Vulnerability factors**							
Belongs to an indigenous group	0	2	0	0	3	3	0	2
Experienced religious/belief discrimination	2	2	1	2	1	4	0	1
Gender and sexual diversity	0	2	0	0	0	0	0	0
Disabilities	0	0	0	0	1	1	0	0
Out of work + 3 mo.	4	5	7	2	3	4	0	0
Government program benefits	2	4	6	2	0	1	2	3
Remittances	0	0	1	0	0	0	0	0

* ES = elementary school; MS = middle school; HS = high school.

## Data Availability

The original contributions presented in this study are included in the article and Supplementary Material. Further inquiries can be directed to the corresponding author.

## Supplementary Materials

The following supporting information can be downloaded at https://www.mdpi.com/article/10.3390/bs16050675/s1, Supplementary Materials S1: Key informant interview (KII) guides and informed consent procedures. Supplementary Materials S2: Focus group discussion (FGD) guides and informed consent procedures. Figure S1: Systems view of the BDM in this study.

## Abbreviations

The following abbreviations are used in this manuscript:

BDM	Behavioral Drivers Model
LAC	Latin America and the Caribbean
SSB	Sugar-sweetened beverage
