# Supplementary material for: Social Norms Around Diet and Body Image: Evidence from Urban and Rural Vulnerable Groups in Colombia and Mexico
Source: Behav Sci (Basel). 2026 Apr 29;16(5):675. doi: 10.3390/bs16050675 (PMC13203662; doi:10.3390/bs16050675)
Supplement: Supplementary file 1 [file behavsci-16-00675-s001.zip › behavsci-4163084-supplementary.pdf]

Supplementary Materials S1. Key informant interview (KII) guides

KII guidelines for government stakeholders

Step 1. Introduction (5 min.)

Introduction of the research team and explanation of the purpose of the research

Script for facilitators to follow:

"Hello, my name is X (name of facilitator), and this is Y (name of assistant facilitator and note-taker). We work at MAGENTA. Thanks for agreeing to take part in the research we are conducting. This study will help us understand the social norms and people's behaviours around nutrition and body image, and to research the factors that drive the consumption of nutritionally poor foods, as well as the rates of overweight. We have invited you to this interview because your professional experience will help us gain more knowledge on the factors that influence what foods people consider to be accessible, appealing and aspirational, in order to ultimately support policies that promote nutrition and health for all people."

Informed consent procedure

Note to facilitators: ensure that each interviewee has signed the consent form included in the project's protocol prior to the start of the interview.

"The document which has been shared with you includes all the details that I will explain to you right now. We are carrying out multiple interviews such as this one to hear from professionals such as yourself, where there is no right or wrong way to answer. Please feel comfortable expressing yourself freely, as all that we talk about here today will stay within this group. When we write the report, we will not mention any names or personal information. Your participation is voluntary, so you have the right to not answer questions and to leave at any time you wish. There are no direct benefits for your participation; however, your views and opinions are very important since UNICEF is working to improve the lives of the people in your community.

"If you do not mind, we would also like to record the discussion because we wish to have a record of the important insights that you share with us, so that we can make sure that we do not miss any points that you make. The audio file would be shared only for research purposes, and we will not share it without your previous consent. However, if you prefer not to be recorded, we will respect your wishes. Furthermore, if you accept to be recorded but then change your mind later during our conversation, we will stop recording immediately. And as you were informed, the discussion will last around one hour. If you agree to participate, please sign two copies of the same document: one copy will stay with me and the other will be given to you."

KII questions for government stakeholders

Step 2. Interviewee's background and experience (5 min.)

"We would like to start this interview with a short introduction from your side. Could you please tell us:

1. What is your general professional background?
2. What is your current professional role? What are your responsibilities?
3. What is the profile of the company/institution/organization you are working for?
4. Which communities and population sectors are you working with?"

Step 3. Views on social norms around nutrition and body image (10 min.)

"The next topic that we would like to cover is about social norms around nutrition and body image. By social norms we are referring to the unspoken rules and expectations about how people act, aim to act, and by which they expect others to act.

5. How would you describe food and drink patterns (nutrition) and food cultures in [your country: either Colombia or Mexico]?
6. How do you define 'healthy' in terms of nutrition and body?
7. How do you think children, adolescents and parents/caregivers in Colombia/Mexico view healthy eating and healthy drinks?
8. How do individuals understand healthy food and drinks? How were these understandings developed?
9. How does the government and/or your agency understand and define 'nutrition' and 'health'? Does this differ at various administrative levels (e.g., federal, state, municipal)?
10. What do you see as the factors that influence nutritional trends in your country?
    I. How do you view, if at all, the relationship between the high consumption of processed foods and of nutritionally poor foods and the context or environmental conditions people live in?
11. What are the structural barriers that influence people's decisions around food? [The following examples can be mentioned if the question is not understood: For instance, have there been increases in food prices, inflation, already long transportation times, electricity and/or water shortages?
12. What do you see as the factors that contribute to trends around overweight?
13. Do you think that views or teachings around overweight have changed over the years? How?"

Step 4. Knowledge, attitudes, and commitments around nutrition and body image (20 min.)

"The next topic we'd like to cover is how your government or governmental agency engages with nutrition and to discuss the opportunities and challenges around that.

14. What factors do you think are the main factors that influence trends around nutrition in your country/region?
    I. How do you see the role of other sectors in influencing nutritional trends?
        1. In particular, what do you see as the role of the private sector in influencing nutritional trends?
15. How does the government/your agency work to influence or counter unhealthy trends around nutrition and body image?
16. What types of programmes are in place, in progress, or absent that promote or hinder nutrition?
17. In particular, what types of programmes are in place, in progress, or absent that promote or hinder the consumption of highly processed foods?
18. What is your government/agency doing to influence these trends, in particular to promote access to nutritious and balanced foods?
    I. How many of these policies/programmes are being or have been implemented?
    II. What are the barriers, if any, to implementing them?
19. Do you think individuals' knowledge and attitudes influence policy implementation? How?"

Step 5. Policies and legislation on diet and body image (15 min.)

"For our final section of the interview, I'd like us to talk about current or previous policies that have encouraged more people to change their behaviour around diet and nutrition.

20. What role does your government play or how does it see its role in promoting nutrition? What types of food policies exist to promote nutrition?
    I. How are these policies designed to work? What is their goal?
    II. Do they target particular populations? If particular, whom do they target and why?
    III. Have these policies been implemented? How do you implement them and ensure compliance?
    IV. How does the government assess and document the state of nutrition in the country? How regularly does this documentation occur?
    V. How, if at all, does this assessment and/or documentation inform policies and programmes aimed at improving nutrition?
    VI. How, if at all, does the government view the relationship between high consumption of processed and nutritionally poor foods and people's environmental settings and context?
21. How does the government and/or your agency view its role, if at all, in informing and educating people about nutrition? What has worked and what you would you consider must be done differently?
22. How would you say that the government has worked around those influences?
23. From your experience, what needs to change first so that more people are consuming fewer processed and nutritionally poor foods? Please give us examples.
24. Are there any specific institutions who are or could also be effective in influencing people's behaviour around nutrition? Which ones and why would they be effective?
25. In your opinion, what would it take to really make a difference in overweight and the consumption of processed and nutritionally poor foods in your country?
26. Is there anything else you would suggest as a step towards changing people's behaviours and decisions?"

Step 6. Closing (5 min.)

- Thank government stakeholder interviewee(s) for their time.
- Tell government stakeholder interviewee(s) where they can get more information about the research later on.
- Ask government stakeholder interviewee(s) if they have any feedback on how the interview was conducted – how could it be improved for the next interviewee(s)?

KII guidelines for advocacy groups, civil society organizations (CSOs) and the media

Step 1. Introduction (5 min.)

Introduction of the research team and explanation of the purpose of the research

Script for facilitators to follow:

"Hello, my name is X (name of facilitator), and this is Y (name of assistant facilitator and note-taker). We work at MAGENTA. Thanks for agreeing to take part in the research we are conducting. This study will help us understand the social norms and people's behaviours around nutrition and body image, and to research the factors that drive the consumption of nutritionally poor foods,
as well as the rates of overweight. We have invited you to this interview because your professional experience will help us gain more knowledge on the factors that influence what foods people consider to be accessible, appealing and aspirational, in order to ultimately support policies that promote nutrition and health for all people."

Informed consent procedure

Note to facilitators: ensure that each interviewee has signed the consent form included in the project's protocol prior to the start of the interview.

"The document I have shared with you includes all the details that I will explain to you right now. As I mentioned, we are carrying out multiple interviews such as this one to hear from professionals such as yourself. Of course, there is no right or wrong way to answer. Please feel comfortable enough to express yourself freely during this conversation, as all that we talk about here today will stay within this group. When we write the report, we will not mention any names or personal information. Your participation is voluntary, so you have the right to not answer questions and to leave at any time you wish. There are no direct benefits for your participation; however, your views and opinions are very important since UNICEF is working to improve the lives of the people in your community.

"If you do not mind, we would also like to record the discussion because we wish to have a record of the important insights that you share with us, so that we can make sure that we do not miss any points that you make. If we need to share the audio file with UNICEF, we will contact you first. We will not share the file without your consent. However, if you prefer not to be recorded, we will respect your wishes. Furthermore, if you all accept to be
recorded but then change your mind later during our conversation, we will stop recording immediately. And as you were informed, the discussion will last around one hour. If you agree to participate, please sign on two copies of the same document: one copy will stay with me and the other will be given to you."

KII questions for advocacy groups, civil society organizations (CSOs) and the media

Step 2. Interviewee's background and experience (10 min.)

"We would like to start this interview with a short introduction from your side. Could you please tell us:

1. What is your general professional background? What is your current professional role? What are your responsibilities?
2. What is the profile of the company/institution/organization you are working for?
3. Which communities and population sectors are you reaching or working with?"

Step 2. Views around diet and body image (10 min.)

"Let's talk about the topic of diet and body image and how

people in Colombia/Mexico view it:

4. How would you describe food and drink patterns (nutrition) and food cultures in [your country: either Colombia or Mexico]?
5. How do you define "healthy" in terms of nutrition and body?
6. How do you think children and adolescents in Colombia/Mexico view and understand healthy foods and drinks?
7. What do you see as the factors that influence nutritional trends in the region?
    I. How do you view, if at all, the relationship between high consumption of processed and of nutritionally poor foods and the context or environmental conditions people live in?
8. What do you see as the factors that contribute to trends around overweight in the region?
9. Do you think views around overweight have changed through the years? How?"

Step 3. Perspectives on advocacy, civil society (CSOs) and media organizations' role in promoting nutrition (15 min.)

"The next topic we would like to cover is the role of your organization in promoting nutrition and/or positive body image for everyone:

10. In what areas does your organization work? What do you see as the relationship or perhaps even the obligation of your organization in promoting nutrition and/or positive body image?
11. What are the structural barriers and opportunities that influence people's decisions around food? [To be mentioned if necessary to clarify question: For instance, have there been increases in food prices, inflation, already long transportation times, electricity and/or water shortages]
12. [If advocacy or civil society organizations:] What types of programmes has, does, or will your organization run to promote nutrition and positive body image?
13. [If media organization:] What types of media programmes or stories has, does, or will, your organization run/broadcast/publish on nutrition and positive body image?
14. What types of opportunities and challenges do you face in implementing these programmes and/or running these stories?
    I. What would make it easier to implement these programmes?
    II. What type of engagement and response do you have from the public/beneficiaries?
    III. What type of engagement and response do you have from other sectors?

1. In particular, what type of engagement, response, support or barriers do you have from the private and public sectors?

15. What do you think would make it possible to change behaviours and trends around the consumption of highly processed and nutritionally poor foods in your country?
16. How do you think we can change social norms around diet and nutrition?
17. What has to be done to positively influence social norms around diet and body image?
18. What are the main reasons that might make people reluctant of healthy nutritional habits?"

Step 4. Narrative and communication channels for nutrition and body image (15 min.)

"Now I would like us to talk about future steps and initiatives that could encourage more people to change behaviour around nutrition and promote both health and positive body image:

19. What type of information is being disseminated around nutrition and body image? By whom?
    I. What is the message of these public forms of information?
    II. What type of institutions have collaborated or delivered information around this?
20. Which platforms have more impact and influence on people's decisions around food?
21. Which campaigns have been successful in positively influencing or increasing access to nutrition? Who implemented them? And why were they successful?
22. In your opinion, what would it take to really make a difference in overweight in your country?
23. Are there any people, organizations, etc., who would be more effective in communicating with people about nutritional food? Who are they and why would they be effective?"

Step 5. Strengths and weaknesses of food environments (10 min.)

"To close our discussion, I'd like us to talk about the strengths and opportunities of food systems and future steps that could encourage more people to change behaviour around diet and body image.

24. How do you think that the availability of processed foods in people's environments influence behaviours around diet and body image?
25. In your view, what has been effective for increasing healthy diets and body image? What has not?
    I. How have private companies thought about those influences?
26. How does the private sector and/or your organization view policy interventions that are aimed at curbing the consumption of processed foods?
27. If the private industry has concerns about food policies, what would it take for the private sector to support policies aimed at curbing the consumption of highly processed foods and beverages?
28. From your perspective, what needs to change in people's environments so that more people make decisions that promote nutrition for themselves? Please give us examples.
29. Are there any specific institutions who are or could also be effective in influencing people's behaviour around diet? Which ones and why would they be effective?
30. Are there any specific private companies in the food sector or in other sectors that in your opinion are influencing people's behaviour around diet and body weight? Could you please list some examples and tell us why do you consider them effective in this regard?
31. Is there anything else you would suggest as a step towards changing behaviours, attitudes, knowledge and social norms around diet and body image/weight?"

Step 6. Closing (5 min.)

- Thank interviewee(s) for their time.
- Tell interviewee(s) where they can get more information about the research later on.
- Ask interviewee(s) if they have any feedback on how the interview was conducted – how could it be improved for the next interviewee(s)?

KII guidelines for the education sector

Step 1. Introduction (5 min.)

Introduction of the research team and explanation of the purpose of the research

Script for facilitators to follow:

"Hello, my name is X (name of facilitator), and this is Y (name of assistant facilitator and note-taker). We work at MAGENTA.
Thanks for agreeing to take part in the research we are conducting. This study will help us understand the social norms and people's behaviours around nutrition and body image, and to research the factors that drive the consumption of nutritionally poor foods,
as well as the rates of overweight. We have invited you to this interview because your professional experience will help us gain more knowledge on the factors that influence what foods people consider to be accessible, appealing, and aspirational in order to ultimately support policies that promote nutrition and health for all people."

Informed consent procedure

Note to facilitators: ensure that each interviewee has signed the consent form included in the project's protocol prior to the start of the interview.

"The document I have shared with you includes all the details that I will explain to you right now. As I mentioned, we are carrying out multiple interviews such as this one to hear from professionals such as yourself. Of course, there is no right or wrong way to answer. Please feel comfortable enough to express yourself freely during this conversation, as all that we talk about here today will stay within this group. When we write the report, we will not mention any names or personal information. Your participation is voluntary, and so you have the right to not answer questions and to leave at any time you wish. There are no direct benefits for your participation, however, your views and opinions are very important since UNICEF is working to improve the lives of the people in your community.

"If you do not mind, we would also like to record the discussion because we wish to have a record of the important insights that you share with us, so that we can make sure that we do not miss any points that you make. If we need to share the audio file with the UNICEF, we will contact you first. We will not share the file without your consent. However, if you prefer not to be recorded, we will respect your wishes. Furthermore, if you all accept to be recorded but then change your mind later during our conversation, we will stop recording immediately. And as you were informed, the discussion will last around one hour. If you agree to participate, please sign on two copies of the same document, one copy will stay with me and the other will be given to you."

KII questions for the education sector

Step 2. Interviewee's background and experience (10 min.)

"We would like to start this interview with a short introduction from your side. Could you please tell us:

1. What is your general professional background? What is your current professional role? What are your responsibilities?
2. What is the profile of the company/institution/organization you are working for?
3. Which communities and population sectors are you reaching or working with?"

Step 3. Views around nutritional diet and body image (15 min.)

"Let's talk about the topic of diet and body image and how people in Colombia/Mexico view it:

4. How would you describe food and drink patterns (nutrition) and food cultures in [your country: either Colombia or Mexico]?
5. How do you define "healthy" in terms of nutrition and body? How does a healthy child or adolescent look like?
6. How do you think children and adolescents in Colombia/ Mexico view and understand healthy foods and drinks?
7. How does the education sector understand and define "nutrition"? Does this differ between different schools or regions?
8. What do you see as the factors that influence nutritional trends in the country?
    I. How do you view, if at all, the relationship between high consumption of processed and nutritionally poor foods and the context or environmental conditions people live in?
    II. What are the structural barriers that influence people's decisions around food? [To mention if the question needs to be clarified: For instance, have there been increases in food prices, inflation, already long transportation times, electricity and/or water shortages]
9. What do you see as the factors that contribute to trends around overweight in the region?
10. Do you think views or teachings around overweight have changed through the years? How?"

Step 4. Views on the education sector's role in influencing behaviours around diet and food (15 min.)

"The next topic we would like to cover is your views on the education sector's role in influencing and informing behaviours around nutrition and food choices:

11. How does the education sector see its role in relation to promoting nutrition for all children?
    I. What types of educational programmes exist to promote nutrition?
       For instance, are there educational programmes? If so, what are they?
       1. How are these programmes implemented?
       2. When and where are the programmes implemented?
       3. What are the curricula of these programmes? How are the curricula developed? Could you share the curricula with us?
       4. Are there privately paid meals at schools and/or publicly funded school meals for children? If so, what are they?
       5. What types of foods are served?
       6. Who decides what to serve? How are decisions made about what to serve?
    II. What are the challenges and opportunities that the education sector sees or has in relation to promoting nutrition?
       For instance, does the sector have informational, awareness or attitude gaps among teachers? How does this affect its delivery of education?
       Are there structural barriers within or outside of the sector that influence the sector's ability to promote nutrition? If so, what are they? How do they influence the sector?

       1. Are these structural barriers uniform across the country? Or do they differ by region, state, district, etc.? If so, how?
12. How does the education sector see its role in promoting positive body image, as well as promoting physical health in children?
    I. How does the sector, if at all, understand and define positive body image and overweight in children?
    II. What types of programmes, if any, exist to promote positive body image and to prevent or address overweight?
       For instance, are there sports or physical education programmes? If so, what are they?
       1. How are these programmes implemented?
       2. When and where are these programmes implemented?
       3. What are the curricula of these programmes? How are the curricula developed? Could you share the curricula with us?
    III. What are the challenges and opportunities that the education

sector sees or has in relation to promoting physical health and positive body image?

Are there structural barriers within or outside the sector that influence the sector's ability to promote physical health and positive body image? If so, what are they? How do they influence the sector?

1. Are these structural barriers uniform across the country? Or do they differ by region, state, district, etc.? If so, how?"

Step 5. Education policies around diet and body image (10 min.)

"To close our conversation, I would like us to talk about current, previous, or potential education policies on food that promote nutrition, physical health, and positive body image for all children:

13. What types of education policies on food exist to promote nutrition for children?
    I. How are these policies designed?
    II. How are these policies implemented?
    Are there policies that exist but that have not been implemented yet? If so, what are they? Why have they not been implemented?
    III. What does "success" look like for these policies? How successful would you say these policies have been, so far?
    IV. What are the challenges and opportunities that influence the success of these policies?
    V. Are there structural barriers that influence the outcomes of these policies?
14. What types of education policies on food exist to promote physical health and positive body image for children?
    I. How are these policies designed?
    II. How are these policies implemented?
    Are there policies that exist but that have not been implemented yet? If so, what are they? Why have they not been implemented?
    III. What does "success" look like for these policies? How successful would you say these policies have been, so far?
    IV. What are the challenges and opportunities that influence the success of these policies?
    Are there structural barriers that influence the outcomes of these policies?
    V. In your opinion, what would it take to really make a difference in promoting nutrition and physical health and preventing overweight in children?
15. What would it take to really make a difference in the rates of consumption of highly processed and nutritionally poor foods in your country?
    I. Are there specific institutions or sectors that could be effective in shifting behaviour and decisions around the consumption of processed and nutritionally poor foods?
    If so, what are they? How do you see their role and influence?
    II. Is there anything else that you would suggest as a step toward changing behaviours around diet, physical health, and body image?"

Closing (5 min.)

- Thank interviewee(s) for their time.
- Tell interviewee(s) where they can get more information about the research later on.
- Ask interviewee(s) if they have any feedback on how the interview was conducted – how could it be improved for the next interviewee(s)?

KII guidelines for the private sector

Step 1. Introduction (5 min.)

Introduction of the research team and explanation of the purpose of the research

Script for facilitators to follow:

"Hello, my name is X (name of facilitator), and this is Y (name of assistant facilitator and note-taker). We work at MAGENTA.
Thanks for agreeing to take part in the research we are conducting. This study will help us understand the social norms and people's behaviours around nutrition and body image, and to research the factors that drive the consumption of nutritionally poor foods, as well as the rates of overweight. We have invited you to this interview because your professional experience will help us gain more knowledge on the factors that influence what foods people consider to be accessible, appealing, and aspirational, in order to ultimately support policies that promote nutrition and health for all people."

Informed consent procedure

Note to facilitators: ensure that each interviewee has signed the consent form included in the project's protocol prior to the start of the interview.

"The document I have shared with you includes all the details that I will explain to you right now. As I mentioned, we are carrying out multiple interviews such as this one to hear from professionals such as yourself. Of course, there is no right or wrong way to answer. Please feel comfortable enough to express yourself freely during this conversation, as all that we talk about here today will stay within this group. When we write the report, we will not mention any names or personal information. Your participation is voluntary, so you have the right to not answer questions and to leave at any time you wish. There are no direct benefits for your participation; however, your views and opinions are very important since UNICEF is working to improve the lives of the people in your community.

"If you do not mind, we would also like to record the discussion because we wish to have a record of the important insights that you share with us, so that we can make sure that we do not miss any points that you make. If we need to share the audio file with UNICEF, we will contact you first. We will not share the file without your consent. However, if you prefer not to be recorded, we will respect your wishes. Furthermore, if you all accept to be recorded but then change your mind later during our conversation, we will stop recording immediately. And as you were informed, the discussion will last around one hour and a half. If you agree to participate, please sign on two copies of the same document, one copy will stay with me and the other will be given to you."

KII questions for the private sector

Step 2. Interviewee's background and experience (5 min.)

"We would like to start this interview with a short introduction from your side. Could you please tell us:

1. What is your general professional background? What is your current professional role? What are your responsibilities?
2. What's the profile of the company/institution/organization you are working for?
3. Which communities and population sectors are you reaching or working with?"

Step 3. Views around nutrition and body image (15 min.)

"Let's talk about the topic of diet and body image and how people in

Colombia/Mexico view it.

4. How would you describe food and drink patterns (nutrition) and food cultures in [your country: either Colombia or Mexico]?
5. How do you define "healthy" in terms of nutrition and body?
6. How do you think children and adolescents in Colombia/Mexico view and understand healthy foods and drinks?
7. How does your industry/company understand and define health? In terms of nutrition? In terms of body?
   I. How does your company promote health and nutrition for staff?
   II. What do you see as the factors that influence nutritional trends in the country?
   III. [If a food & beverage company:] Who are your main customers and audiences?
   IV. [If a food & beverage company:] What do customers like about your products?
      1. How do you keep customers satisfied?
      2. How do you keep customers engaged?
      3. How do you think about what makes foods appealing and aspirational?
8. Who are your main consumers and audiences of your company and products?
9. Within the private sector, in particular in the food and beverage industry, how do these parts of the private sector view health and the status of metabolic health in your country?
10. What does your company/institution/organization see as their role in promoting health and nutrition in your country?
11. What are the structural barriers that influence people's decisions around food? For instance, have there been increases in food prices, already long transportation times, electricity and/or water shortages?
12. What do you see as the factors that contribute to trends around overweight in the region?
13. Do you think views or teaching around overweight have changed through the years? How?"

Step 4. Attitudes and commitment around diet and body image (15 min.)

"The next topic we would like to cover is your views on the private sector's role in influencing and informing behaviours around nutrition and food choices:

14. What do you understand by healthy diet or healthy eating patterns (HEP)? What do you think society in general understands about them?
15. What factors do you think are the main drivers of social norms in the country/region?
16. How do private companies influence these social norms around diet, nutrition and body image? How about other actors?
17. How aware do you think people are of healthy diet or healthy eating patterns and their benefits? What drives/motivates them to follow HEP or not?
18. Do you see any obstacles to HEP? What is the source of these obstacles in your opinion?
19. Do you think there are any misconceptions around healthy nutritional habits? Which ones and how do they affect people? How do you think these misconceptions were developed?
20. What do you think has to be done to promote healthy eating patterns and change unhealthy eating patterns¿ Please consider psychological, sociological and environmental factors. Who needs to do it? [Facilitators could explain the factors and dimensions within each of the three levels.]
21. How do you think we can change social norms around diet and nutrition in children, adolescents and in their parents/caregivers?
22. What must be done to positively influence social norms around diet and body image?
23. What are the main reasons that might make people reluctant of healthy nutritional habits?"

Step 5. Strengths and weaknesses of food environments (15 min.)

"To close our discussion, I would like us to talk about the strengths and opportunities of food systems and future steps that could encourage more people to change behaviour around diet and body image.

24. How do you think that the availability of processed foods in people's environments influence behaviours around diet and body image?
25. In your view, what has been effective for increasing healthy diets and body image? What has not?
   I. How have private companies thought about those influences?
26. How does the private sector and/or your company view policy interventions that are aimed at curbing the consumption of processed foods?
27. If the private industry has concerns about food policies, what would it take for the private sector to support policies aimed at curbing the consumption of highly processed foods and beverages?
28. From your perspective, what needs to change in people's environments so that more people make decisions that promote their own nutrition? Please give us examples.
29. Are there any specific institutions who are or could also be effective in influencing people's behaviour around diet? Which ones and why would they be effective?
30. Are there any specific private companies in the food sector or in other sectors that in your opinion are influencing people's behaviour around diet and body weight? Can you please list some examples, and tell us why are you considering them effective in this regard?
31. Is there anything else you would suggest as a step towards changing behaviours, attitudes, knowledge and social norms around diet and body image/weight?"

Step 6. Closing (5 min.)

- Thank private sector interviewee(s) for their time.
- Tell private sector interviewee(s) where they can get more information about the research later on.
- Ask private sector interviewee(s) if they have any feedback on how the interview was conducted – how could it be improved for the next interviewee(s)?

Supplementary Materials S2. Focus group discussion (FGD) guides

FGD guidelines for mothers, fathers and/or caregivers of children aged 0–5 years, and children and adolescents aged 5–19 years

Introduction (5 min.)

INSTRUCTION	SCRIPT
Introduce yourself and your assistant.	"Hello everybody, my name is X (name of moderator) and this is Y (name of assistant moderator and note-taker). We work at MAGENTA. Thanks for agreeing to take part in this research."
Outline the study back- ground and FGD purpose.	"We are trying to find out about social norms around food and eating.<br>"We are interested in knowing how people eat, cook, and share meals in your community. We are having conversations with people in many communities across Latin America to paint a picture about the cultures of food and eating in the region.<br>"UNICEF is supporting this research and the results of our research will be published as a report."
Explain the ethical consent procedure.	"Prior to this session, each of you was given a document entitled 'Letter of consent' with the objectives of this session, in which we informed you how we will use this information, and the important points about the privacy of your name and personal data. Do you have any questions about the content of that document?"<br><br>[MODERATORS: If doubts arise, clarify them according to the guidelines contained in the consent letter.]
Lay down the ground rules for the discussion.	"Before we start, I want to highlight the following points about the dynamic of the session:<br>1. This session will last between 90 and 105 minutes (1:30 and 1:45 hours). It is a group conversation: the idea is that we talk to each other and share our views.<br>2. My job as moderator is to ask the questions and make sure that everyone has the opportunity to participate.<br>3. Please keep your microphone on silent during the session, to prevent external noise and be sure we can always hear each other.<br>4. To participate, all you have to do is point at the camera or activate the "Raise your hand" button. Please don't speak at the same time as another person or interrupt them while they are speaking.<br>5. There are not correct or incorrect answers or ways to answer.<br>6. All your opinions and experiences are very important to us and we want to hear them.<br>7. No one is to be judged here.<br>8. If you agree, I will start recording the session when I start the conversation, after we introduce ourselves."
Focus the discussion on the age of participants' children.	"I ask you, please, that when we talk about issues related to the nutrition of your sons or daughters, we focus on your sons or daughters under 5 years of age / sons or daughters between 6 and 19 years of age (according to the group's profile)."

Warm up (5 min.)	
**Q#**	**Question**
**Note to moderators**: The left column entitled "Question" shows the primary research question that we are aiming to answer in each section. The column to the right, entitled "Probes", shows the supplementary questions. We are interested in answering these questions as much as possible to provide a holistic picture on eating and food; however, the list is lengthy. It is therefore not necessary to ask each and every question. Please treat the probes as a guide for robust discussion.	
**1**	"Please introduce yourself, tell us your name or the name by which you would like to be called by in this discussion, and tell us the age of your children as well. It doesn't have to be your real name if you don't want to."<br>[Note to MODERATORS: Start the recording at the end of the introductions.]

Family's eating habits (20 min.)		
**Q#**	**Question**	**Probes**

**2**	"What do you usually eat on a typical day?"	• "How many and what meals do you eat on a typical day? What do you like to eat, typically? [Note to MODERATORS: from there, ask about each moment/meal.]<br>• For example, what did you have for breakfast yesterday? And your children?<br>• What did you eat in the afternoon? And your children?<br>• What did you have for dinner? And your children?<br>• And between meals, what did you and your children eat?"<br>• "What foods or snacks do you commonly eat when you have cravings? At what times of the day or the week does that happen?<br>• How many and what types of vegetables and fruits do you eat on a typical day?<br>• What types of beverages do you regularly drink and how often?<br>• How often do you drink water, juice, or soda?<br>• How often do you drink home-made beverages, such as tea and coffee? If you add sugar, how much?<br>• How often do you go out to eat or buy food on the street?<br>When you eat on the street, do you eat in restaurants, cafes or street stalls, or do you bring your own food from home?"
**3**	"What types of pastries, sweets, and candies do you like?"	□ "How many times per week do you eat these foods and sweets?<br>□ Where do you usually get them? Do you make them?<br>□ If you buy them, from where?"
**4**	"If you like to drink soda, what type do you like to have?"	"When you drink soda (carbonated sugar-sweetened beverages), what types do you like to have?<br>□ Do you have favourite brands of soda?<br>□ How many times a week/day do you drink soda or soft drinks? Do you drink soda with your meals (e.g., with breakfast, lunch, dinner)?<br>□ When your child or children have soda or soft drinks, what types do they like to drink?<br>□ How old was your child or were your children when he/she had a soda for the first time?<br>□ How many times a week/day does your child or do your children drink soft drinks?
	**Purchases or ways of obtaining food (15 min.)**	
**Q#**	**Question**	**Probes**
**5**	"Where do you regularly buy or get the food ingredients for your family meals?"	• "Do you grow any food at home? Do you raise animals?<br>• Where do you get the foods that are most common in your home? For example, tortillas, rice, beans, *arepas*, bread, etc.<br>• Where do you regularly get your fruits and vegetables?<br>• Which products do you buy the most?<br>• Where do you regularly buy snacks/treats and desserts?<br>• Are there times when it's hard to afford the food you want or need to buy?<br>• How far do you or your family has to travel and what transport do they use to get the food they buy?"
	**Food preparation and meal timing (20 min.)**	
**Q#**	**Question**	**Probes**
**6**	"How do you decide what to cook for your children/what is cooked for your children on a typical day? Whether you are responsible for cooking or someone else."	[Note to MODERATORS: Please remind participants to focus on their sons or daughters under 5 years of age / sons or daughters between 6 and 19 years of age (according to the group's profile).<br>□ "How do you or someone in your household decide what to cook?<br>□ What types of foods do your children eat for their meals and for snacks?<br>□ How many and what types of vegetables and fruits do they eat on a typical day?<br>□ What types of beverages do your children drink and how often?<br>□ What role do women and men have in making meals?"
**7**	"How is food prepared at home for your family?	□ "How is food prepared at home?<br>□ Are all meals prepared at home? Which are not?<br>□ What is the role of children and adolescents in the preparation of food at home?"

**8**	For mothers / fathers / guardians of young children:] "How do you feed or fed your baby or toddler?"	• "Do you or did you breastfeed your children?<br>• How was your experience?<br>• How did you decide to breastfeed?<br>• How did you decide to stop breastfeeding?<br>• When and how did you start incorporating other foods into your children's diet?<br>• What types of foods and drinks did you introduce to your children? For example, they started with porridge or mashed foods, broths and soups, teas, etc.<br>• What ingredients were or are included in those foods and drinks?<br>• How do you think these ingredients help the health of your babies or young children?"
**9**	"How is food served to your children? How are they fed? How do your children react?"	• "Once you have cooked and the food is ready, how is the food served at home?<br>• What is the role of children in serving food?<br>• How would you say your children eat?<br>• What happens when you try a dish with vegetables that your children have not tried before or do not usually eat? What do you tell them to encourage them to try these new or unusual ingredients?<br>• If they dare to try these new or unusual ingredients but then say that they are full, what do you do or what do you tell them?<br>• Do any of your reactions change if it is a girl or a boy who does not want to finish the dish or does not want to try the food? If so, how do your reactions change and why do you think the situation is different?"
**Activity**		
**10. Social network mapping**	Guiding questions for moderators' reference [do not read aloud to participants]: Who are the people in participants' social circles? How does information about food and health flow in their social circles? The moderator of each group discussion will be provided with a diagram of the Social-Ecological Model, which the moderator will fill out based on the information that participants provide.<br><br>The Social-Ecological Model's diagram will depict five concentric circles, each of which represents an increasingly distant part of a person's community: the centre circle represents the individual; the second circle represents their family; the third one represents their peers; the fourth represents members of their broader community; and the fifth represents the media (e.g., television, radio, etc.). This diagrammed network enables researchers to both analyze how information is shared within a community and which forms of information are important to them.<br><br>Instructions:<br>"1. Let's think about the past week or month and focus on the conversations you have had in that period of time with your **family** about carbonated sugar-sweetened beverages, that is, sodas (for instance, Coca-Cola) and junk food, or about food in general. Please just focus on your family for now.<br>"What type of conversations about sodas and junk foods have you had with your family?<br>What type of sodas and junk foods do members of your family drink and eat? When do you or anyone in your family have sodas and junk food (e.g., at meals, at parties, with guests)?<br>Do you or anyone in your family drink soda when you don't feel well? Why? What effects does it have? How did you learn about this remedy?<br>What impact do soda and junk food have on one's health?<br>Would you say that this is a difficult topic to discuss with your family or that it is easy to talk about this with them<br><br>"2. Now let's think about your **friends** and your **closest circle**, that is, the people with whom you interact the most outside of your own family, such as your neighbors, co-workers or other parents from your children's school.<br>• What type of conversations about sodas and junk foods have you had with your friends and closest circle?<br>• What type of sodas and junk foods do your friends and members of your closest circle drink and eat? When do they have sodas and junk food (e.g., at meals, at parties, with guests)?<br>• Do any of your friends or members of your closest circle drink soda when they don't feel well? Why? What effect do they say it has? How did they learn about this remedy?<br>• What impact do your friends or members of your closest circle say that soda and junk food have on their health?<br>• Would you say that this is a difficult topic to discuss with your friends and members of your closest circle, or that it's easy to talk about this?"	

	"3. Now let's go a little further, beyond family or friends, to include people from your **community**. For example, it can be a neighborhood leader, a religious leader, teachers, even the owner of the store or market stall, or the cashier at the supermarket. Perhaps these conversations are less common, but let's think in general about these types of conversations with other people in your community.<br>What type of conversations about sodas and junk foods have you had with people in your community?<br>How do people in your community talk about sodas and junk food? How important are sodas and junk food in your community?<br>Would you say that this is a difficult topic to discuss with people in your community, or that it's easy to talk about this?"<br><br>"4. Finally, let's now think about the **media**, since we also obtain information about food through television programmes, on the radio, on social networks, in the newspaper, even in advertisements that we see on the street, painted on a wall, on billboards or in public transport and buses. Let's please think of concrete examples, that is, instead of talking about something you saw "on television", tell me the name of the television programme and channel/broadcaster or even the person who appears in that programme which for you is an important source of information about food.<br>• Where and when have you seen information about soda and junk food recently?<br>• What was it about?<br>• Did you learn something you didn't know?<br>• Was it only an advertisement or do you also remember seeing advice or recommendations?"	
**11.**	"Which of these circles has the greatest influence on and relevance for you?"	• "Of these four social circles, which is the one that really influences you the most when deci- ding what foods to buy and/or cook?<br>• Please briefly share an example with us."<br>• After the conversation is complete, thank everyone for their participation:<br><br>"Thank you very much for doing this activity with us. This information is going to be very helpful for us.<br>"Is there anything else that anyone would like to add before we move on?"

FGD Guidelines for adolescents aged 14–16 years

Introduction (5 min.)

Instruction	Script
Introduce yourself and your assistant.	"Hello everybody, my name is X (name of moderator) and this is Y (name of assistant moderator and note-taker). We work at MAGENTA. Thanks for agreeing to take part in this research."
Outline the study background and FGD purpose.	"We are trying to find out about social norms around food and eating.<br><br>"We are interested in knowing how people eat, cook, and share meals in your community. We are having conversations with people in many communities across Latin America to paint a picture about the cultures of food and eating in the region.<br><br>"UNICEF is supporting this research, and the results of our research will be published as a report.<br><br>"We think that your views are very important, and we all hope that this study will inform future projects to improve nutrition and health for all children in Latin America."
Explain the ethical consent procedure.	[• Confirm the signing of the consent letter:]<br>"Prior to this session, each of you and your fathers, mothers or guardians were given a document to read, entitled 'Letter of consent', with the details and objectives of this session, and from what we understand, both you and your fathers, mothers or guardians have already signed it in agreement with it. Is there anyone who hasn't signed it yet? Do you have any questions about the content of that document?"<br><br>[MODERATORS: If doubts arise, clarify them according to the guidelines contained in the consent letter. If someone has not signed the letter of consent, please proceed and ask them, as well as the father, mother or guardian, to sign it online. If they don't sign it, they can't participate in the group.]<br>"Just to confirm, can you please tell me your age? [Please ask everyone. Participants must be between 14 and 16 years of age; if someone is younger, they cannot participate in the session.]
Refer to ESOMAR sensitive topic discussion guidelines among young people (14-16 years old).	A detailed description about what is going to be asked and about the purposes of the study must to be given to both the parent and the child. Their agreement must be obtained to continue.

Lay down the ground rules for the discussion.	"Before we start our discussion, I want to make sure we set some ground rules:<br><br>"1. WE WANT YOU TO DO THE TALKING. We would like everyone to participate. We highly encourage everyone to share their views. All your opinions and experiences are very important and we want to hear them.<br>"2. THERE ARE NO RIGHT OR WRONG ANSWERS. Everyone's experiences and opinions are important. Speak up whether you agree or disagree. We want to hear from all of you. No one will be judged here.<br>"3. WHAT IS SAID ON THIS PLATFORM STAYS HERE. We want everyone to feel comfortable sharing their opinion regarding any issues that come up. When we write our report, we will not mention any name or personal information of any of you.<br>"4. WE WILL NOT TALK AT THE SAME TIME. We want to take turns to talk, so we can all hear your opinions and experiences. Feel free to express yourself freely during the discussion, as long as you do it respectfully.<br>"Would you like to add any additional rules?<br>"Is everything clear about the course of the discussion?"<br>[If everyone says things are clear, proceed with the discussion. If not, make sure to answer all inquiries and questions before starting the discussion.]<br><br>The discussion will last between one and a half and two hours (between 90–120 minutes).<br>If you want to stop the discussion for any reason, please just raise your hand and I will be happy to do so. If any questions that I ask you make you feel uncomfortable, you can ask me to skip them. If someone changes their mind during the session, or has any questions, feels uncomfortable or even wants to stop participating, please let us know.
Reiterate confidentiality and ask participants to agree to same principles.	"Before we begin, I want to remind you that your participation is voluntary and that if anyone wishes to stop the conversation at any point and wants to stop participating, please feel free to tell me. If any questions that I ask you make you feel uncomfortable, you can ask me to skip them. If anyone has any questions, please let us know.<br><br>"However, we hope that you will stay and share your opinions with us, since they are very valuable in our mission to improve the living conditions of the people of your country, especially that of children, adolescents, their fathers, mothers and caregivers.<br><br>"If you agree, we also want to record this conversation, since there are several of us in this session and it would be impossible to be able to take notes of all their opinions or remember everything, they told us afterwards. Can we record the session when we start talking?<br><br>"I remind you, no one else will have access to that conversation other than the researchers who are participating."<br><br>"The discussion today may be quite sensitive and even personal. For this reason, we will not share the recording with anyone outside the project team. We will not write your names in any of the notes. If there is anything you say that you don't want us to write down, let us know after the discussion and we will delete it.<br><br>"We are committed to protecting you and the information you share with us. We want you to feel safe."

**Warm up (5 min.)**	
**Q#**	**Questions**
	**Note for moderators**: The left column entitled "Question" shows the primary research question that we are aiming to answer in each section. The column to the right, entitled "Probes", shows the supplementary questions. We are interested in answering these questions as much as possible to provide a holistic picture on eating and food; however, the list is lengthy. It is therefore not necessary to ask each and every question. Please treat the probes as a guide for robust discussion.
1	Please introduce yourself, tell us the name you would like to be called by in this discussion. It doesn't have to be your real name if you don't want to. Please also share with us if you go to school and, if that is the case, in which grade you are.<br>[Note to MODERATORS: Start the recording at the end of the introductions.]

Home		
**Food Habits (20 min.)**		
**Q#**	**Question**	**Probes**
**2**	"What do you usually eat on a typical day?"	• "How many and what meals do you eat on a typical day? What do you like to eat, typically?" [Note to MODERATORS: from there, ask about each moment/meal.]<br>o "For example, what did you have for breakfast yesterday?<br>o What did you eat in the afternoon?<br>o What did they have for dinner?<br>o And between meals, what did you eat?"<br>• In addition to all that you ate yesterday, what other foods are common in your daily life?"[Note to MODERA- TORS: explore at different times of the day, not just at the main meal.]<br>• "What foods or snacks do you commonly eat when you have cravings? At what times of the day or the week does that happen?<br>• How many and what types of vegetables and fruits do you eat on a typical day?<br>• What types of beverages do you regularly drink and how often?<br>• How often do you drink water, juice, or soda?<br>• How often do you drink home-made beverages, such as juice, tea, coffee, or another beverage? If you add sugar, how much?<br>• How often do you go out to eat or buy food on the street?<br>• When you eat on the street, in what type of place do you usually eat: inn, street stall, restaurant, store food?<br>• And when it comes to a special meal, what things change from what you've mentioned so far? Tell me, what foods are special to you? What makes them special? When and with whom do you eat these foods?"
**3**	"What types of sweet foods and candy do you like?"	• "How many times a week do you eat these foods and sweets?<br>• Where do you usually get them from? Do you make them? Do you buy them? Where?"
**4**	"If you like to drink soda, what type of soda do you like to drink?"	• "When you have soda, what types of soda do you like to have?<br>• Do you have favourite brands of soda?<br>• How many times per week or per day do you drink soda? Do you drink soda with your meals (for instance with breakfast, lunch, or dinner)?"
**Buying or other form of getting food in the family (5 min.)**		
**Going out to eat**		
**5**	"How does your family get food and ingredients for your meals?"	• "Who buys the ingredients for your meals?<br>• Is there any food that you grow at home? Do you breed animals?"<br>[Note to MODERATORS: if they say that they buy meals or ingredients themselves, ask:]<br>• "What do you look for in the food and the ingredients that you buy?<br>• [For the participant or anyone in their family:] "Do you think that sometimes it is difficult to obtain foods that you consider are necessary to buy? Why?<br>• How far do you have to travel and what transport do you use to acquire the food you buy?
**6**	"Where do you normally buy or get food for your family?"	• "Where do you get the foods that are most common in your home? For example, tortillas, rice, beans, *arepas*, bread, etc.<br>• Do you have a favourite brand or type? For example, corn tortillas vs wheat flour tortillas.<br>• Where do you regularly get your fruits and vegetables?<br>• Which ones do you buy the most?<br>• Where do you regularly get your sauces, pickles and condiments?<br>• Do you have any favourite brands?<br>• Where do you regularly buy snacks and desserts?<br>• Do you have any favourite brands?"
**7**	"How do people in your community eat?"	• "Think about what you cook, what you eat, and where you buy it: how different or similar is the way people in your community eat compared to the way you eat at home?<br>• Where do people in your community buy foods and ingredients for meals?<br>• How far do they have to go? What transport would you say they use?"

**8**	"When you eat outside your home, where do you usually eat?"	• "When you eat outside your home, where do you usually eat?<br>• When or for what reason do you eat outside of your home?<br>• With whom do you usually go?<br>• What are the places that you most like to go to eat? Which ones do you like but don't go as often or just can't go at all?<br>• Who would you like to go with?"

Food preparation and mealtimes (10 min.)

Q#	Question	Probes
**9**	"How do you prepare foods at home in your family?"	• "What is your role in preparing foods at home?<br>• Do you know how to cook? How did you learn to cook?"
**10**	"How do you add flavour? Do you add flavour while you're cooking food or after?"	• "Do you add any kind of flavouring, seasoning or condiment to your food?<br>• For example, do you normally add salt, spices or sugar to your food? To which? Why?"
**11**	"How is food served at your home?"	• "Once your food has been cooked and is ready, how is it served at your home?<br>• What is your involvement in serving food?<br>• When do you eat?<br>• How do you eat?<br>• How much are you expected to eat?"

Activity	Activity instructions (20 min.)
**12. Social network mapping (based on the Social-Ecological Model)**	Guiding questions for moderators' reference [do not read aloud to participants]: Who are the people in participants' social circles? How does information about food and health flow in their social circles? The moderator of each group discussion will be provided with a diagram of the Social-Ecological Model, which the moderator will fill out based on the information that participants provide.<br><br>The Social-Ecological Model's diagram will depict five concentric circles, each of which represents an increasingly distant part of a person's community: the centre circle represents the individual; the second circle represents their family; the third one represents their peers; the fourth represents members of their broader community; and the fifth represents the media (e.g., television, radio, etc.). This diagrammed network enables researchers to both analyze how information is shared within a community and which forms of information are important to them.<br><br>**Instructions:**<br>"1. Let's think about the past week or month and focus on the conversations you have had in that period of time with your family about carbonated sugar-sweetened beverages, that is, sodas (for instance, Coca-Cola) and junk food, or about food in general. Please just focus on your family for now.<br><br>"What type of conversations about sodas and junk foods have you had with your family?<br>What type of sodas and junk foods do members of your family drink and eat? When do you or anyone in your family have sodas and junk food (e.g., at meals, at parties, with guests)?<br>Do you or anyone in your family drink soda when you don't feel well? Why? What effects does it have? How did you learn about this remedy?<br>What impact do soda and junk food have on one's health?<br>Would you say that this is a difficult topic to discuss with your family or that it is easy to talk about this with them?"<br><br>"2. Now let's think about your friends and your closest circle, that is, the people with whom you interact the most outside of your own family, such as your neighbors, classmates, co-workers (if you already have a job), the parents of your friends and classmates.<br><br>What type of conversations about sodas and junk foods have you had with your friends and closest circle?<br>What type of sodas and junk foods do your friends and members of your closest circle drink and eat? When do they have sodas and junk food (e.g., at meals, at parties, with guests)?<br>Do any of your friends or members of your closest circle drink soda when they don't feel well? Why? What effect do they say it has? How did they learn about this remedy?<br>What impact do your friends or members of your closest circle say that soda and junk food have on their health?<br>Would you say that this is a difficult topic to discuss with your friends or members of your closest circle, or that it's easy to talk about this?"

	"3. Now let's go a little further, beyond family or friends, to include people from your community. For example, it can be a neighborhood leader, a religious leader, teachers, even the owner of the store or market stall, or the cashier at the supermarket. Per- haps these conversations are less common, but let's think in general about these types of conversations with other people in your community.<br><br>What type of conversations about sodas and junk foods have you had with people in your community?<br>How do people in your community talk about sodas and junk food? How important, if at all, are sodas and junk food in your community?<br>Would you say that this is a difficult topic to discuss with people in your community, or that it's easy to talk about this?"<br><br>4. Finally, let's now think about the media, since we also obtain information about food through television programmes, on the radio, on social networks, in the newspaper, even in advertisements that we see on the street, painted on a wall, in billboards or in the public transport and buses. Let's please think of concrete examples, that is, instead of talking about something you saw "on television", tell me the name of the television programme and channel/broadcaster or even the person who appears in that programme which for you is an important source of information about food.<br><br>Where and when have you seen information about soda and junk food recently? What was it about?<br>Did you learn something you didn't know? Was it only an advertisement or do you also remember seeing advice or recommendations?"	
**13**	"Which of these circles has the greatest influence on and relevance for you?"	"Of these four social circles, which is the one that really influences you the most when deciding what foods to buy and/ or cook? Please briefly share an example with us."
**14**	"What does 'healthy' mean to you?"	"What types of foods do you and people in your family, your circle of friends and acquaintances and your community consider to be healthy and not healthy?"
	After the conversation is complete, thank everyone for their participation:<br><br>"Thank you very much for doing this activity with us. This information is going to be very helpful for us. "Is there anything else that anyone would like to add before we move on?"	
**Activity**	**Body appreciation (15 min.)**	
**15. Participants will fill out a short survey, to be explored via a reflective conversation.**	"Now we are going to do another activity that will allow us to explore some ideas about body image. It is a survey that you will answer from your cell phone or computer.<br>"The survey is very short, just seven questions about your ideas on health and body image. When you answer, please do so in the blank space that corresponds to each question on the survey.<br>"Please remember, there are no right or wrong answers to these questions. Answer what you think: that is the most valuable response for this study.<br>"My partner is going to share a link in the chat of our online meeting. Please click on it and tell me when you are ready. The question *'Please enter the group number indicated by your moderator'* should appear onscreen.<br>"To answer it please use the group number I am going to give each of you individually."<br>[Note to the MODERATOR: check that all participants are able to open the survey, tell each participant the group number that they must register to enter and ask them to click the green button to start the survey.]<br>"Did everyone enter the survey yet? Does anyone have a problem?"<br>[Note to the MODERATOR: show the figure that illustrates the first question or show how the survey appears on your cell phone, and explain the instruction and answer bar for question 1.]<br>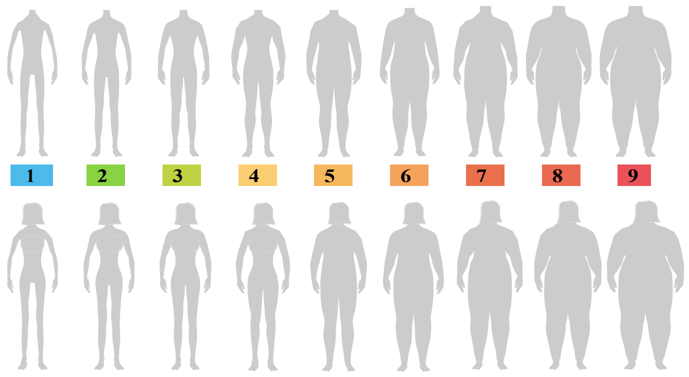<br>Figure S1. Stunkard Figure Rating Scale (Stunkard, Sorensen, and Schulsinger, 1983).82	

	"The image on the screen shows nine different pairs of drawings of men and women with different sizes and body shapes. Can everyone see it?<br>"Question 1) says: What is the drawing that best represents the healthiest body for women? Below is a bar with numbered buttons from 1 to 9. Choose your answer and simply touch the corresponding number button.<br>"Does anyone have a problem to access it? Any other questions?<br>"Now please continue by answering the questions that will appear on your screen at your own pace. There are six questions in total:<br>1. What is the drawing that represents the healthiest body for women? Select the number of the corresponding figure.<br>2. What is the drawing that represents the healthiest body for men? Select the number of the corresponding figure.<br>3. Considering the drawings you chose to represent the healthiest body for women and the healthiest body for men, do you think there are differences between the two figures? What differences do you find between them?<br>4. What is the drawing that best represents how women are seen in your community? Select the number of the co- rresponding drawing.<br>5. What is the drawing that best represents how men are seen in your community? Select the number of the corres- ponding drawing.<br>6. From your point of view, what would be the three main ways to achieve a healthy body? "Keep responding at your own pace, I'll wait for you here, let me know as y4ou finish.<br><br>"Just make sure you click the last green button at the end of the survey to send us your answers."

**Wrap-Up (5 min.)**		
**16**	"How do you think that health in your community and/or country could be improved?"	• "What things do you consider help or make it difficult for all children and adolescents in your commu- nity and/or country to have access to healthy nutrition and body image?<br>• What types of approaches do you think could help adolescents in your community and/or country to have access to nutrition and health?"
**17**	Final thoughts	"Is there anything else you would like to add to this FGD?" [If yes, please take notes.]<br>• Thank participants for their time.<br>• Tell participants where they can get more information about this research later on.<br>• Ask participants if they have any feedback on how the discussion was conducted – what could improve the discussion for the next group?
